# Anomalous coherent and dissipative coupling in dual photon-magnon hybrid resonators

**DOI:** 10.1038/s41598-024-64315-x

**Published:** 2024-06-12

**Authors:** Haechan Jeon, Bojong Kim, Junyoung Kim, Biswanath Bhoi, Sang-Koog Kim

**Affiliations:** Department of Materials Science and Engineering, National Creative Research Initiative Center for Spin Dynamics and Spin-Wave Devices, Nanospinics Laboratory, Research Institute of Advanced Materials, Seoul National University, Seoul, 08826 Republic of Korea

**Keywords:** Materials science, Physics

## Abstract

We explored the distinctive behavior of coherent and dissipative photon-magnon coupling (PMC) in dual hybrid resonators, each incorporating an Inverted Split-Ring Resonator (ISRR) paired with a Yttrium Iron Garnet (YIG) film, positioned in close proximity but with varying relative split-gap orientations. These orientations led to notable shifts in the dispersion spectra, characterized by level repulsion and attraction, signaling coherent and dissipative coupling, respectively, in single ISRR/YIG hybrids at certain orientations. Through analytical modeling, we determined that the observed shifts in coupling types are primarily due to the effect of photon-photon (ISRR-ISRR) interactions altering the phase difference between the coupled ISRR and magnon modes. Our findings highlight that precise manipulation of the relative split-gap orientations in the ISRR resonators enables controlled coherent and dissipative coupling within planar PMC systems. This capability opens new avenues for applications in quantum information technologies and quantum materials.

## Introduction

The interplay between light and matter constitutes a cornerstone of physics with pivotal applications across quantum optics, cavity quantum electrodynamics, and telecommunications^1–3^. A particularly intriguing form of light-matter interaction is the coupling between photons and magnons—the quanta of electromagnetic microwaves and collective spin excitations, respectively. Earlier studies on photon-magnon coupling (PMC) predominantly focused on coherent coupling, manifested through level repulsion in dispersion and level attraction in linewidth^4–7^. This phenomenon stems from a distinct phase relationship between the photon and magnon modes, a consequence of an interaction between spin ensembles and electromagnetic fields^8^. Coherent coupling facilitates transduction across different systems, enabling controlled energy exchange and signal transfer.

Conversely, dissipative coupling represents another vital facet of light-matter interactions, characterized by energy dissipation within a system due to its interaction with the environment^9–12^. Investigations by M. Harder et al.^13^ and B. Bhoi et al.^14^ into dissipative coupling within a three-dimensional (3D) cavity and an inverted split-ring resonator (ISRR)/YIG hybrid system, respectively, underscore its significance. Given its implications for developing nonreciprocal devices, such as optical isolators, and enhancing sensing techniques, dissipative coupling has garnered considerable attention^13,15–19^. In quantum systems, it can precipitate coherence loss and entanglement degradation, highlighting the importance of managing both coherent and dissipative couplings to bolster quantum system stability and fidelity, paving the way for advanced quantum information technologies^20^.

In this study, we engineered dual PMC hybrids comprising a photon (ISRR) and a magnon resonator interconnected via a microstrip line, with the ISRRs arranged in varying relative split-gap orientations. This setup yielded disparate dispersion spectra, characterized by both level attraction and repulsion, in the dual ISRR/YIG hybrid, contingent on the relative split-gap orientation. By employing analytical modeling and comparing it with experimental observations, we unraveled the anomalous coupling behaviors of each YIG/ISRR hybrid. Analysis revealed that interactions between photon-photon hybrid modes and the magnon mode—and the resulting phase differences—can substantially modify the typical coherent and dissipative PMC dynamics observed in singular hybrid systems, due to photon-photon (ISRR-ISRR) interactions. Our findings elucidate the complex dynamics of multi-photon interactions within PMC systems and facilitate the integration of spin degrees of freedom into photonic systems, thereby advancing the application of photon-magnon coupling research.

## Results

### Dual photon resonators composed of two ISRRs

**Figure 1:**
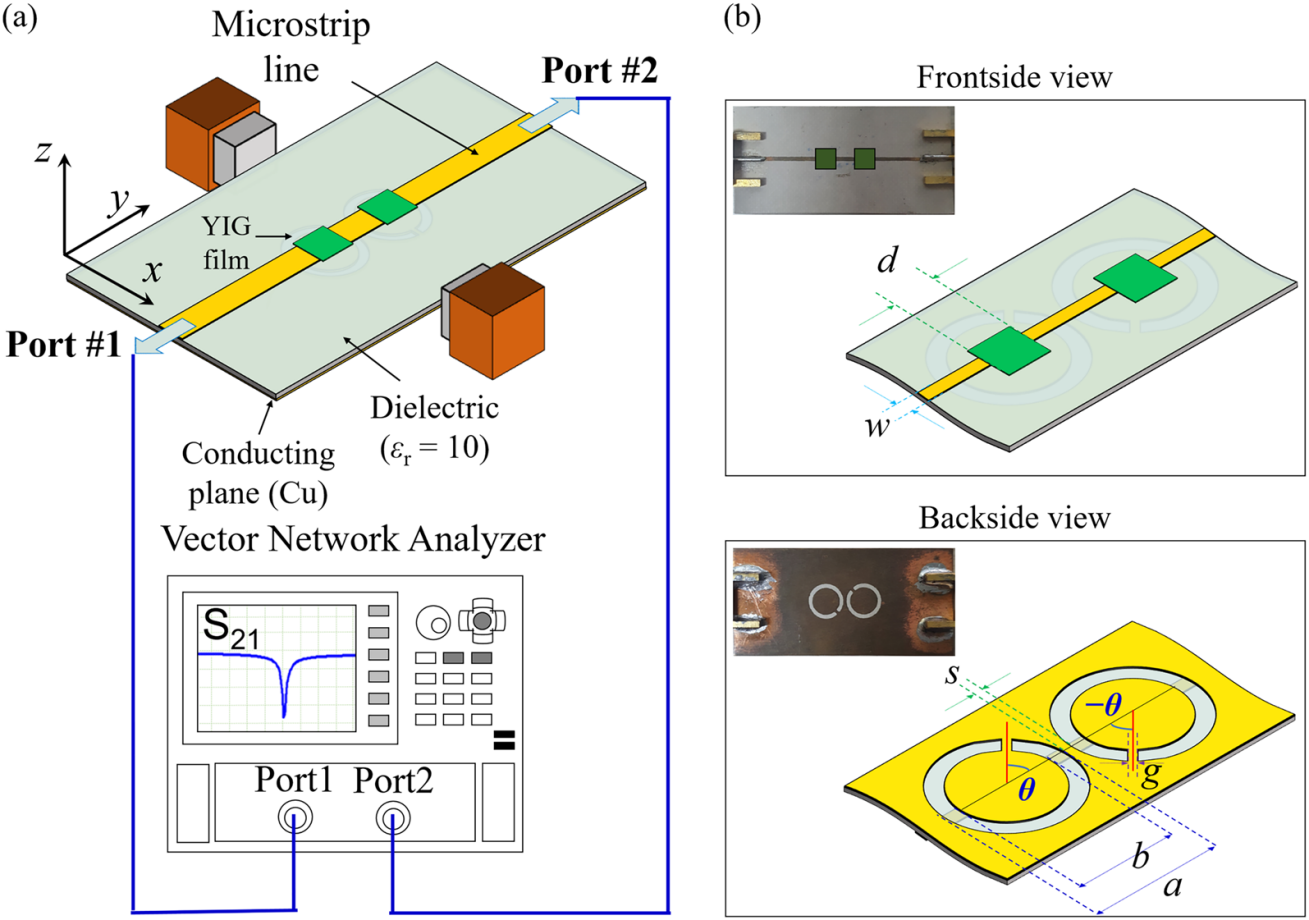
(**a**) Schematic of the measurement setup for scattering parameters in a dual YIG/ISRR hybrid system. (**b**) Schematic of the sample geometry with optical images showing front (top) and rear (bottom) views of the sample. The two ISRRs are positioned closely, mutually influencing their dynamic magnetic and electric fields. Identical YIGs are positioned on opposite sides at each ISRR’s center. The orientation of the split-gap, *θ*, is defined as the angle of the split-gap position relative to the microstrip-line axis, as illustrated at the bottom of (**b**). As *θ* increases, the gap position of the left ISRR rotates counterclockwise, whereas that of the right ISRR rotates clockwise. All the dimensions of the YIG, ISRR, and microstrip line are provided in the Methods section.

**Figure 2:**
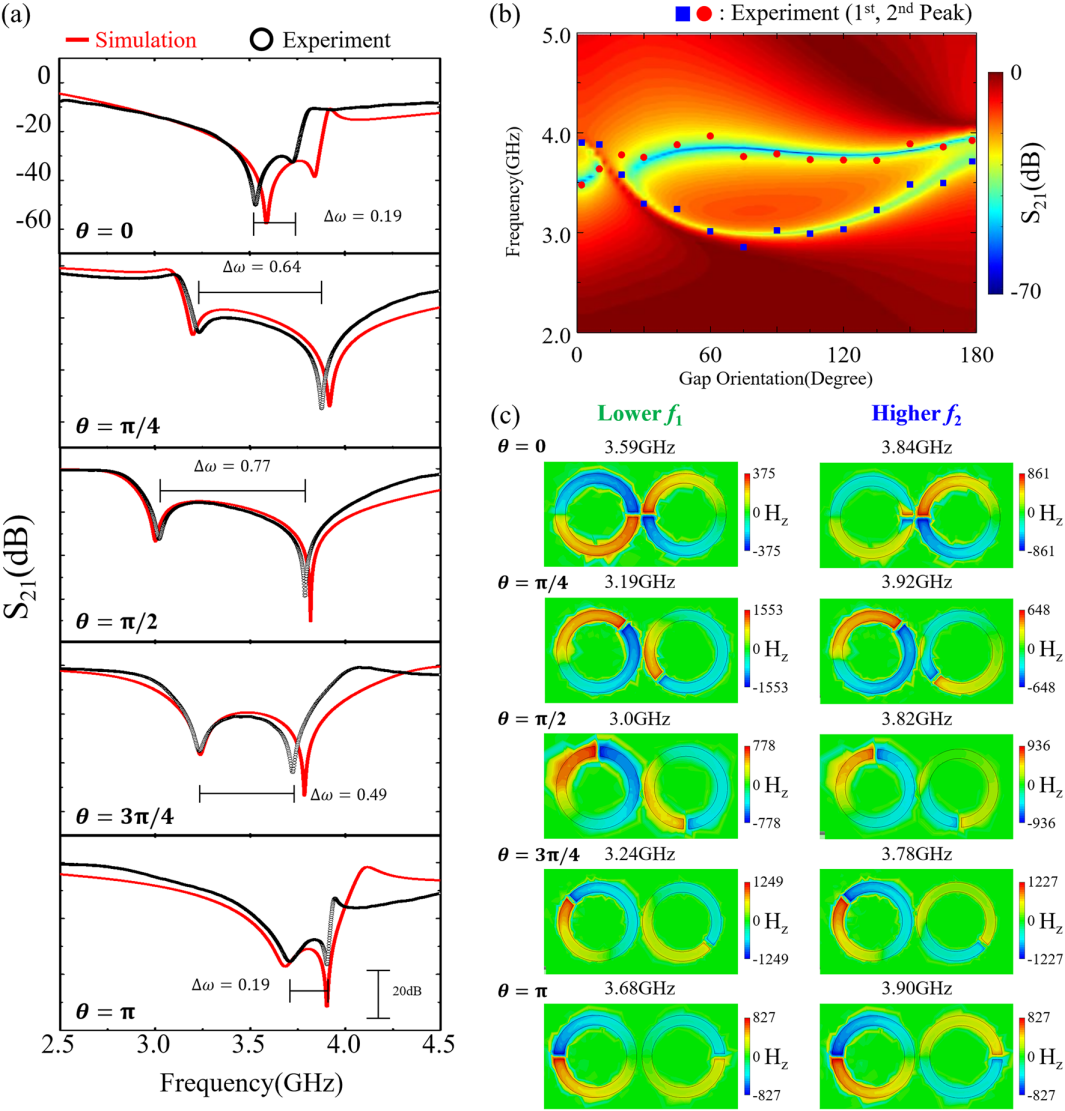
(**a**) Comparison of S_21_ power spectra from simulations (red lines) and experimental observations (black open circles) for only the two ISRRs without YIG films. The peak splits of the dual ISRRs’ coupled modes, Δω/2π, are marked with horizontal lines in the experimental data. (**b**) Contour plots of the simulated S_21_ power spectra across the AC frequency and the split-gap angle (*θ*), compared with the experimental data (represented by closed symbols) for various specified angles. Red circles and blue squares correspond to the eigenfrequencies of the higher- and lower-branch modes, respectively. (**c**) Spatial distributions of the our-of-plane magnetic field (**H**_z_) at the resonance frequencies indicated above each image.

Figure 1a displays our measurement setup for the S_21_ scattering parameter, featuring two ISRRs and two yttrium iron garnet (YIG) films (indicated by green squares) alongside a standard microstrip line (depicted as an orange wide line). The two identical YIG films are strategically placed at the center of each ISRR on opposite sides, as depicted in Fig. 1b. The placement of the YIG films at the center of each ISRR is critical because photon-magnon dynamics are significantly influenced by the distance between the YIG and ISRR. This strategic positioning minimizes the physical variability associated with the YIG location, thereby enhancing the effective observation of photon-photon interactions. We optimized the ISRRs' dimensions and geometry through numerical electromagnetic wave simulations using CST Microwave Studio^7^. The orientation angle of the split-gap, *θ*, relative to the microstrip line axis (y-axis), was adjusted to values of *θ* = 0, *π*/4, *π*/2, 3*π*/4, and *π*, as illustrated at the bottom of Fig. 1b. This relative split-gap orientation influences the hybrid modes' eigenfrequencies in the dual ISRRs, observable in the S_21_ power spectra (black lines) measured exclusively from the two ISRRs without YIG films, as shown in Fig. 2a. The simulation results (red lines) closely align with the experimental spectra regarding the modes' eigenfrequencies. Any minor discrepancies between the experimental and simulation findings can result from impedance mismatches due to the coaxial-to-microstrip line transitions at the connectors' ends and dimensional inconsistencies between the actual samples and the modeled ISRRs.

Figure 2b presents the contour plot of S_21_ power spectra versus frequency across various split-gap angles *θ*, ranging from 0 to *π* in increments of *π*/36, as predicted by CST studio simulations. From the experimental findings from Fig. 2a, the resonant frequencies of the lower- and higher-branch hybrid modes are denoted by blue squares and red circles, respectively. The alignment between experimental and simulated data underscores the accuracy of the simulations, which notably reveal that at *θ* = *π*/12, the eigenfrequencies of the higher and lower hybrid photon modes converge, as noticed by vertical arrow. This intersection is traditionally associated with level attraction, indicative of dissipative coupling within diverse photon systems^21^.

To understand deeper into the photon-photon hybridized modes and their dependency on the relative split-gap orientation, we display snapshot images of the spatial distributions of the out-of-plane magnetic field (Hz) at specific resonance frequencies of the coupled photon modes, as derived from CST studio simulations. These visualizations highlight the dynamic attributes of the photon hybrid modes. In the dual-ISRR configuration, the hybrid photon modes are discerned based on their multipolar symmetry^22^, with a consistent observation that the out-of-plane fields are oppositely directed across the split gaps in each resonator, mimicking magnetic dipoles—except for the higher mode at *θ* = 0 (top right in (c)). The Hz distributions display quadrupole configurations near the gap proximity, featuring four magnetic nodes, and dipole configurations with two magnetic nodes^23^.

The ${\mathbf{H}}_{\text{z}}$ component in Fig. 2c demonstrates a diminishing effect on higher-order photonic excitations as the split-gap angle *θ* varies^22^. This attenuation, evidenced by changes in the ${\mathbf{H}}_{\text{z}}$ amplitude, can be theoretically elucidated by the mutual inductance between the dual ISRRs. As the spatial separation of the two split gaps increases, the mutual inductance—critical for magnetic interaction between the ISRRs—diminishes, leading to a decrease in high-order photon-photon interaction. Consequently, this decrease results in a convergence of the ${\mathbf{H}}_{\text{z}}$ amplitude at the two resonance frequencies, indicative of a more uniform photonic excitation. This interpretation is corroborated by the ${\mathbf{H}}_{\text{z}}$ components in Fig. 2c, derived from the Maxwell equations^24,25^. Moreover, at $\theta =0$, the maximum ${\mathbf{H}}_{\text{z}}$ component exhibits a significant amplitude difference, with 375 A/m at the lower and 861 A/m at the higher resonance frequencies, marking a disparity of 2.3 times. This divergence persists at $\theta =\pi /4$, with values of 1553 A/m and 648 A/m, indicating a 2.4 times difference. Yet, as $\theta$ values increase, an intriguing pattern emerges: the magnetic field strengths for the two resonance modes begin to harmonize, ultimately converging at $\theta =\pi$ to a uniform strength of 827 A/m. This pattern suggests the waning impact of higher-order interactions with increasing $\theta$ values. Thus, this effect delineates a distinction between dipolar and quadrupolar photonic interactions, which are intrinsically linked to photon-photon interactions, especially in the cases of $\theta =0$ and $\pi /4$.

To comprehend this interaction between photon modes, we consider the hybridized states arising from the coupling of two undisturbed photon modes^26,27^. Among these, the lower mode aligns with the photon's first-order resonance or base mode (n = 0), while the alternative state, the higher mode (n > 1), corresponds to the hybrid higher mode aligned with the second-order resonance^24,25,28^. Typically, the dipole configuration is viewed as the most basic photon resonance mode, whereas quadrupolar photonic excitation is interpreted as stemming from a second-order interaction. The subsequent analysis will explore how direct coupling between the two ISRRs influences each type of photon-magnon coupling, whether coherent or dissipative.

### Dual hybrid resonators, each composed of YIG/ISRR and alternations of photon-magnon coupling

**Figure 3:**
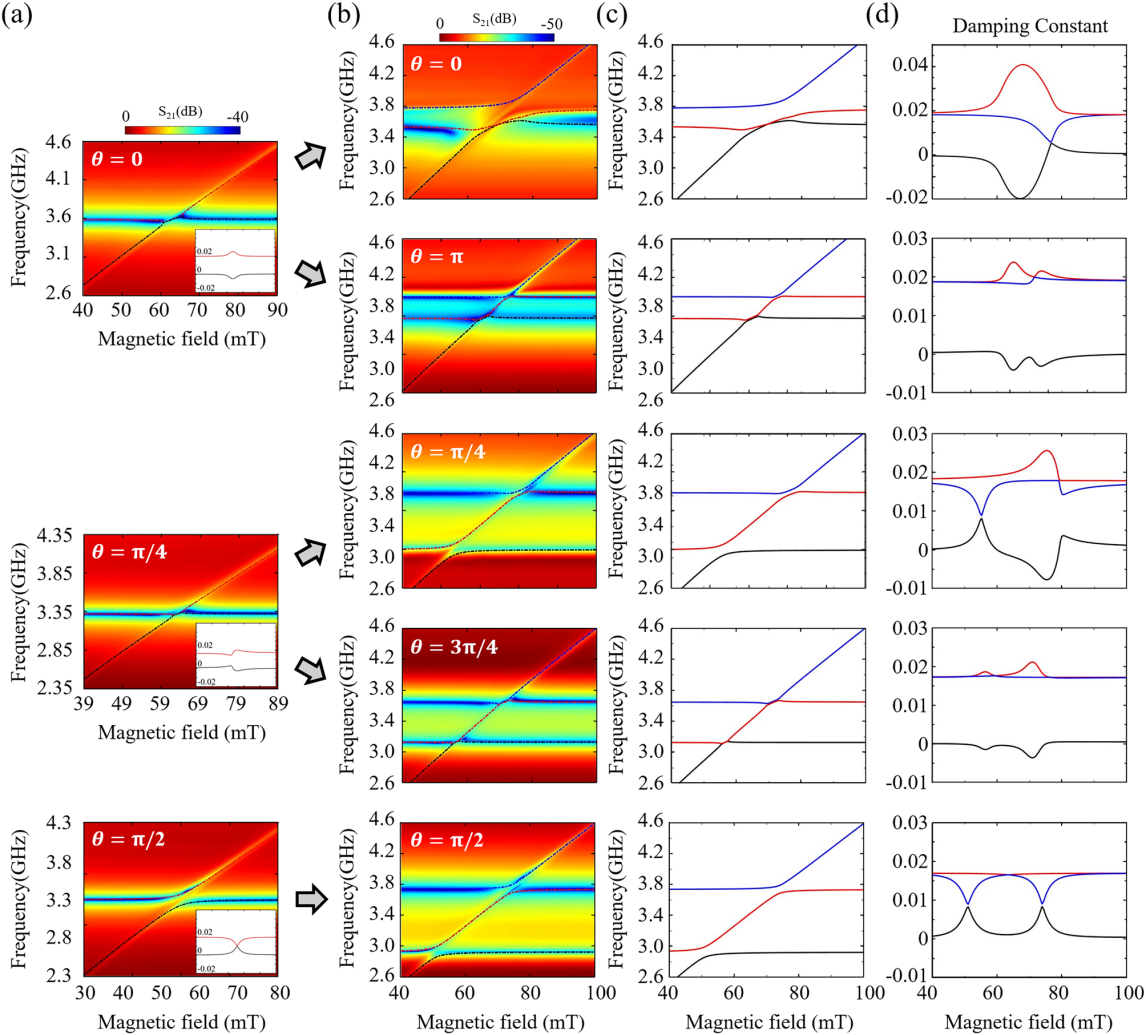
(**a**) Experimentally measured S_21_ power spectra for single ISRR/YIG samples at *θ* = 0, $\pi /4$, and $\pi /2$. The damping constants for each case are presented as insets within the plot. (**b**) Experimentally measured S_21_ power spectra for dual YIG/ISRR samples with varying relative split-gap orientation: *θ* = 0, $\pi$, $\pi /4$, $3\pi /4$, and $\pi /2$. (**c**) Real and (**d**) imaginary components of the complex eigenfrequencies for three hybrid modes for the dual YIG/ISRR hybrids, derived from the analytical model described in the text. In (**c**), the first, second, and third hybrid modes are represented by the blue, red, and black lines, respectively. In (**d**), the magnon-dominant mode is denoted by the black line.

Building upon the photon-photon interactions within the two ISRRs, we conducted experimental measurements of the S_21_ power spectra for dual ISRR/YIG hybrids, as illustrated in Fig. 3b, compared with single ISRR/YIG hybrid samples as shown in 3(a). In single YIG/ISRR hybrids at $\theta =0, \pi /4$, and $\pi /2$, we noted patterns of level repulsion at $\theta =\pi /2$ and level attraction at $\theta =0$, in alignment with findings from our previous report^7^. Notably, at $\theta =\pi /4$, we found a blend of both level attraction and repulsion, appearing as mixed crossing. While the coupling dispersion of $\theta =\pi /4$ did not change significantly in dissipative coupling, the ratio between the real and imaginary parts of the eigenvalue changes as the split gap orientation changes. Specifically, at $\theta =\pi /4$, there is a larger real part compared to $\theta =0$, and a larger imaginary part compared to $\theta =\pi /2$. These trends are well illustrated in the inset of Fig. 3a, which depicts the damping constants within a single photon-magnon hybrid system; here, the black line represents the photon’s damping constant, and the red line denotes that of the magnon. The damping constant changes, dependent on the type of coupling, allows us to infer the direction of energy transfer between the two components. Particularly, at $\theta =0$, the direction of energy transfer is biased, resulting in the magnon's damping reaching the negative region. In this scenario, the decay time of the magnon increases as dissipation decreases, leading to more stable and prolonged excitation. A thorough elucidation of these phenomena's origins and a detailed account based on the split-gap angle are available in our earlier work^14^.

As illustrated in Fig. 3b, dual ISRR/YIG hybrids at $\theta =0$, $\pi$, $\pi /4$, $3\pi /4$, and $\pi /2$ demonstrate varied dispersions in photon-magnon couplings, influenced by $\theta$. Each YIG film is coupled with its corresponding photon resonator ISRR, exhibiting either level repulsion or attraction at the coupling between the magnon and photon modes, with the specific nature of these interactions contingent upon $\theta$. This variation suggests that photon-photon interactions between the ISRRs modify the PMC, leading to either coherent (level repulsion) or dissipative (level attraction) coupling. The dotted lines in Fig. 3b denote three hybrid modes derived from analytical modeling, to be discussed subsequently. To better illustrate these modes, we replot the real components of the complex eigenfrequencies for the three hybrid modes in Fig. 3c, using three distinct colors to represent each mode. These modes display level attraction and/or repulsion at shared resonance frequencies between the two singular ISRR modes (indicated by two horizontal lines) and the magnon mode (illustrated by a single diagonal line).

In detail, at $\theta$ = 0, PMC at the lower frequency (3.53 GHz) exhibits level attraction, whereas at the higher frequency (3.73 GHz), level repulsion is apparent. At $\theta =\pi$, level attraction is observed at both the lower (3.71 GHz) and higher (3.90 GHz) frequencies. For $\theta =\pi /4$, level attraction at the higher frequency (3.88 GHz) and level repulsion at the lower frequency (3.23 GHz) are noted. At $\theta =3\pi /4$, there is level attraction—or possibly mixed coupling—at both the lower (3.23 GHz) and higher (3.72 GHz) frequencies. Conversely, at $\theta =\pi /2$ , clear level repulsion is noted at both the lower (3.02 GHz) and higher (3.79 GHz) frequencies. The dispersion patterns at $\theta =\pi /4$, $3\pi /4$, and $\pi$ resemble those observed in single ISRR/YIG hybrids with corresponding split-gap orientations, as depicted in Fig. 3a. However, an unusual pattern of level repulsion at the higher frequency is observed at $\theta =0$. Similarly, at $\theta =\pi /4$, the presence of level repulsion at the lower frequency deviates from the expected behavior of PMC in a single YIG/ISRR hybrid at the same angle, as illustrated in the middle of Fig. 3a. The mixed coupling dispersions at $\theta =3\pi /4$, indicating a transitional phase between level attraction and repulsion, align with the behavior seen in a single YIG/ISRR hybrid at $\theta =\pi /4$, as shown in the middle of Fig. 3a. The diverse dispersion types identified in dual YIG/ISRR hybrids cannot be solely explained by the coupling dynamics observed in single YIG/ISRR hybrids at equivalent split-gap orientations^4^. Despite identical split-gap orientations between the two ISRRs as in single ISRR configurations, the PMC dispersions in dual hybrids exhibit variations due to the photon-photon (ISRR-ISRR) interaction affecting each magnon-photon (YIG-ISRR) coupling.

Utilizing Eq. (3) from the analytical modeling section, we fitted the three experimentally observed hybrid modes presented in Fig. 3b. This process enabled the estimation of the damping constants for these modes, illustrated in Fig. 3d. The calculated damping constants elucidate the observed phenomena of level attraction and repulsion at both lower- and higher-frequency regions, including instances of anti-damping (indicated by negative values of damping constants). This observation aligns with the presence of crossing and anti-crossing patterns in the linewidths between the two hybrid modes at shared resonance frequencies, correlating with coherent and dissipative coupling, respectively. When examining the three hybrid modes for $\theta =0$ and $\pi$, the scenario at $\theta =\pi$ reveals a repulsion in linewidths between the magnon-dominant mode (black lines) and the photon-dominant modes (blue and red lines), indicative of typical dissipative PMC. Conversely, at $\theta =0$, significant anti-damping, up to $\alpha =-0.02$, was observed at the lower resonance frequency (3.53 GHz), with linewidth crossing behavior noted at the higher frequency (3.73 GHz). This suggests extensive anti-damping across a broad lower-frequency range. For $\theta =\pi /4$ and $\theta =3\pi /4$, both attraction and repulsion patterns in the linewidths mirror those in single hybrids, contingent on the split-gap orientation. At $\theta =\pi /2$, the linewidth profile resembles that of coherent coupling observed in single photon-magnon systems, where minimal nonlinearity occurs as the imaginary part of the complex eigenvalue approaches zero, behaving akin to a Hermitian system^29^. Consequently, energy exchange between the systems manifests as typical coherent coupling, evidenced by the crossing of linewidths^26^.

### Analytical modeling

To understand the influence of photon-photon interaction on the photon-magnon coupling type within each photon/magnon hybrid, we employed an analytical model based on a multiphoton-magnon Hamiltonian framework that incorporates coupling dynamics between particle-bosonic modes and cavity field excitations^30^. In this model, the interaction within each photon-magnon coupling mirrors the interplay between a two-level atom and a solitary field excitation source^31^. The Hamiltonian for our system is designed to capture three hybrid modes—two photon-dominant and one magnon-dominant—reflecting the dual photon-magnon couplings in the YIG/ISRR hybrids and a lone photon-photon interaction across the two ISRRs^26^. Within these hybridized states, the photons and magnons exhibit significant phase disparities^32–34^. Thus, the Hamiltonian^35^ is formulated as follows:

1 $$H_{JC} = \omega_{1} a^{\dag } a + \omega_{2} b^{\dag } b + \omega_{m} S_{z} + g_{1} \left( {aS_{ + } + a^{\dag } S_{ - } } \right) + g_{2} \left( {bS_{ + } + b^{\dag } S_{ - } } \right) + g_{P} \left( {ab^{\dag } + a^{\dag } b} \right)$$

where *a*^†^(*a*) and *b*^†^(*b*) are the creation (annihilation) operators for both photon resonators. The Pauli operators $S$_+_ and $S$_–_ describe the interactions between spins, while ${S}_{z}$ denotes the z-component of the spin operator. The term ${\upomega }_{\text{m}}$ is the resonance frequency of the magnon mode*.* The constants ${g}_{\text{1,2}}$ indicate the coupling strength between each photon and the magnon mode in each YIG/ISRR hybrid, and ${g}_{P}$ denotes the coupling strength between the two proximately positioned ISRRs. These three coupling constants are described by the following equations:

2a $${g}_{P}={k}_{0}+{k}_{1}cos\theta +{k}_{2}{cos}^{2}\theta +{k}_{3}{cos}^{3}\theta$$

2b $${g}_{1}={J}_{1}+{\Gamma }_{1}\mathit{cos}\left(\theta +{\phi }_{M1}\right)+i{\Gamma }_{1}sin(\theta +{\phi }_{M1})$$

2c $${g}_{2}={J}_{2}+{\Gamma }_{2}\mathit{cos}\left(\theta +{\phi }_{M2}\right)+i{\Gamma }_{2}sin(\theta +{\phi }_{M2})$$

where $\theta$ represents the split-gap orientation angle relative to the microstrip line, playing a crucial role in determining the photon-magnon interactions. The phase differences between the magnon mode and each hybrid photon mode are denoted by ${\phi }_{M1}$ and ${\phi }_{M2}$. The terms ${J}_{\text{1,2}}$ and ${\Gamma }_{\text{1,2}}$, represent the coherent and dissipative coupling constants for each photon-magnon coupling, respectively. The constant ${g}_{P}$ is expressed as a series of cosines of *θ*^28^, stemming from the multipole expansion illustrated in Eq. (2a). The constants ${k}_{0},{k}_{1} ,{k}_{2} ,{k}_{3}$ correspond to the amplitude of each multipolar excitation. They remain fixed for the dual-photon-magnon hybrid system under investigation and are obtained from fitting the to the eigenfrequency variations observed experimentally across five different dual ISRRs with varying *θ*. The results are as follows:${k}_{0}=486\text{MHz},{k}_{1} =169\text{MHz},{k}_{2}=-448\text{MHz} ,{k}_{3}=-402\text{MHz}$. Based on these values, the amplitude of each multipolar excitation can be manipulated by varying the gap angle orientation $\theta$.

The complex eigenfrequencies of the three hybridized modes thus can be determined using the following matrix;

3 $$\mathit{det}(H-\omega \Gamma )=\left(\begin{array}{ccc}n{\omega }_{1}+m{\omega }_{2}+{\omega }_{m}-\omega & {g}_{1}\sqrt{n+1}& {g}_{2}\sqrt{m+1}\\ {g}_{1}\sqrt{n+1}& {\omega }_{1}(n+1)-\omega & {g}_{p}\sqrt{(n+1)(m+1)}\\ {g}_{2}\sqrt{m+1}& {g}_{p}\sqrt{(n+1)(m+1)}& {\omega }_{2}(m+1)-\omega \end{array}\right)$$

where ${\omega }_{\text{1,2}}$ is the resonance frequency of each photon mode*.* The mode numbers of the photon resonators are presented by n and m, with n and m being zero for the lowest base modes. The presence of non-zero diagonal terms in the matrix indicate active participation of all components in the hybridized coupling. To account for intrinsic damping, ${\omega }_{1}$ is replaced with ${\widetilde{\omega }}_{1}={\omega }_{1}-i{\beta }_{1} ,{\omega }_{2}$ with ${\widetilde{\omega }}_{2}={\omega }_{2}-i{\beta }_{2}$ *,* and ${\omega }_{m}$ with ${\widetilde{\omega }}_{m}={\omega }_{m}-i\alpha$, where $\alpha$*,* ${\beta }_{\text{1,2}}$ are the damping constants of the magnon and each photon modes, respectively. By solving Eq. (3)*,* the complex eigenfrequencies of the three hybrid modes, ${\widetilde{\omega }}_{a}$ ${\widetilde{\omega }}_{\pm }$*, *${\widetilde{\omega }}_{-}$*,*are determined as follows:

$${\widetilde{\omega }}_{a}=\frac{1}{3}\left\{\left({\widetilde{\omega }}_{1}+{\widetilde{\omega }}_{2}+{\widetilde{\omega }}_{m}\right)+(A+B)\right\}$$

4 $${\widetilde{\omega }}_{\pm }=\frac{1}{3}\left\{\left({\widetilde{\omega }}_{1}+{\widetilde{\omega }}_{2}+{\widetilde{\omega }}_{m}\right)-\frac{1}{2}(A+B\pm \sqrt{3} i(A-B)\right\}$$

with $$A, B=\sqrt[3]{\frac{1}{2}({X}^{2}\pm \sqrt{{X}^{2}-4{Y}^{3}}}$$

where $\text{X and Y}$ are given as

5a $$\text{X}={\left(2{\omega }_{ISRR}+{\omega }_{m}\right)}^{3}-3\left(2{\omega }_{ISRR}^{3}+{\omega }_{m}^{3}\right)+15{\omega }_{ISRR}^{2}{\omega }_{m}+\left({\omega }_{m}-{\omega }_{ISRR}\right)\left({g}_{1}^{2}+{g}_{2}^{2}+{g}_{p}^{2}\right)+6{g}_{1}{g}_{2}{g}_{p},$$

5b $$Y={\left({\omega }_{m}-{\omega }_{ISRR}\right)}^{2}-3\left({g}_{1}^{2}+{g}_{2}^{2}+{g}_{p}^{2}\right),$$

Assuming ${\widetilde{\omega }}_{1}={\widetilde{\omega }}_{2}$ as the single ISRR frequency due to the coupled ISRR being identical, the coupling constants ${g}_{\text{1,2}}$ between each photon hybrid mode and magnon mode can be obtained by fitting to the experimentally measured three hybrid modes in dual ISRR/YIG hybrids with different split gap orientations shown in Fig. 3c.

### Influence of photon-photon interaction on coherent and dissipative photon-magnon coupling

**Figure 4:**
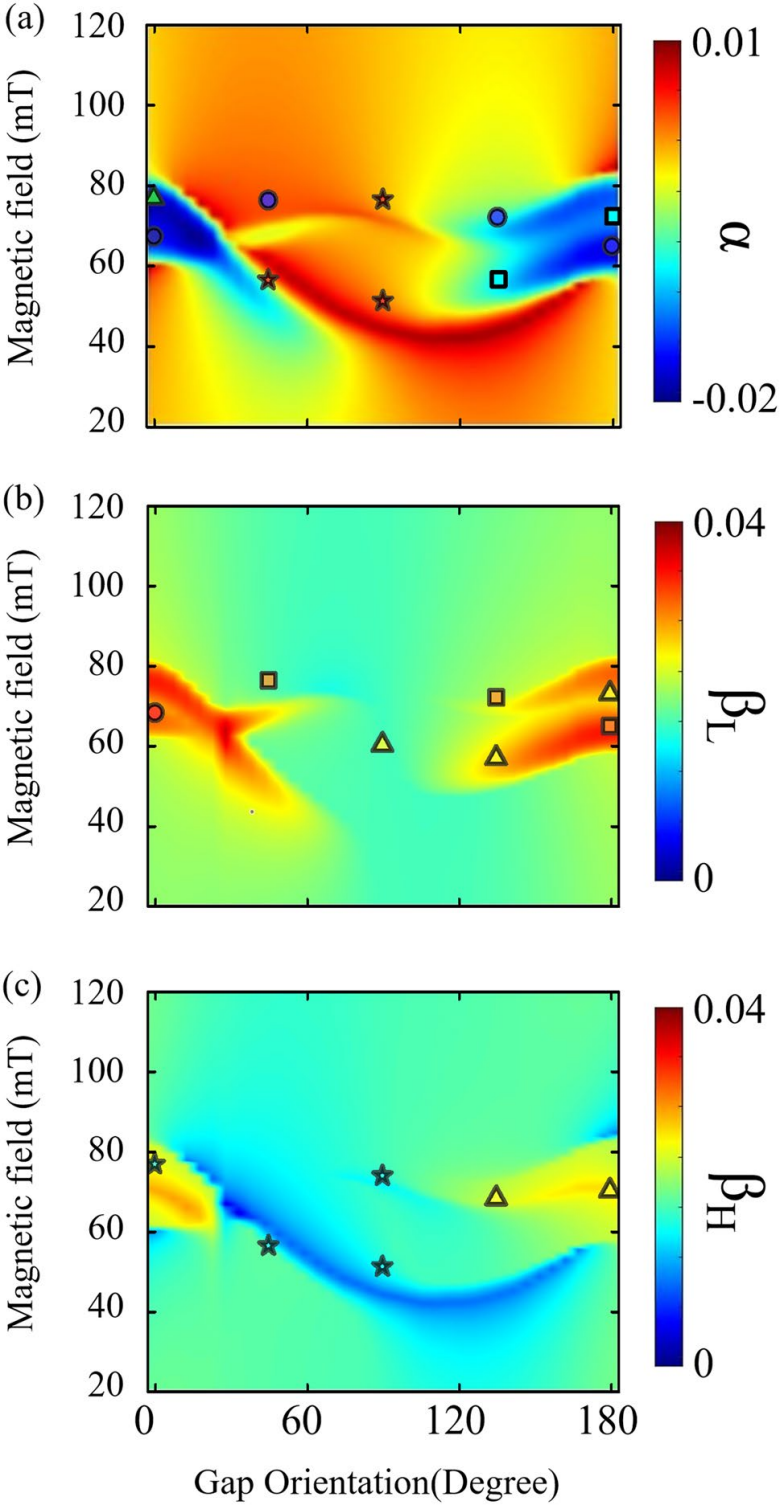
Analytical and experimental damping constants for multi-photon-magnon coupling in magnon-dominant and photon-dominant hybrid modes. This figure presents the analytical calculation of damping constants, with ${\phi }_{M1}{= \phi }_{M2}=\uppi /2$. Experimental values from multi-photon-magnon coupling (refer to Fig. 3b), represented by symbols in various shapes. The colors of the symbols correspond with the 2D contour plot's color variation. The colormap gradient represents the magnitude of damping, providing a visual correlation between experimental data and theoretical predictions. (**a**) Experimental data points are symbolized as circles (*α* ≈ − 0.02), squares (*α* ≈ − 0.01), triangles (*α* ≈ 0), and stars (*α* ≈ 0.01) on 2D contour plot of calculated magnon damping constant. The anti-damping is strongly revealed in low and ultra-high theta regions. (**b**) and (**c**) show the damping constants ${\beta }_{\text{L}}$ and ${\beta }_{\text{H}}$ for each photon-dominant hybrid modes, indicated by circles ($\beta \sim 0.04$), squares ($\beta \sim 0.03$), triangles ($\beta \sim 0.02$), and stars ($\beta \sim 0.01$), highlighting the damping variations across different gap angle.

In our investigation of photon-photon interaction effects, we determined the damping constants of the magnon-dominant mode across a range of *θ* from 0 to *π*, especially when ${\phi }_{M1}{=\phi }_{M2}$ is set to $\pi /2$, as depicted in Fig. 4a. Notably, the blue region, indicative of negative damping and suggestive of dissipative coupling, spans a wide range of field strengths from $\theta =0$ to $5\pi /36$ and from $2\pi /3$ to $\pi$. In contrast, positive damping values, signifying coherent coupling, are predominantly observed from $\theta =\pi /6$ to $5\pi /9$, as shown by the red region. These experimental observations underscore dynamic shifts in damping based on *θ*, diverging from the damping behaviors seen in single ISRR-YIG couplings, as addressed in earlier publications^13,14,32^. Furthermore, Fig. 4b,c show the damping constants for the hybrid photon modes, revealing a marked increase in positive damping (indicative of energy loss) within the level attraction regions compared to the intrinsic damping of photon modes, defined as ${\beta }_{i} =0.02$. Within the same *θ* range of 0 to $5\pi /36$ and $2\pi /3$ to $\pi$, an enhancement in negative *α* values was observed alongside increased positive $\beta$ values. This distinctive trend is attributed to shifts in the relative intensities of the photon-photon hybridized modes as a function of *θ*, leading to variations in dispersion type within each photon-magnon coupling.

**Figure 5:**
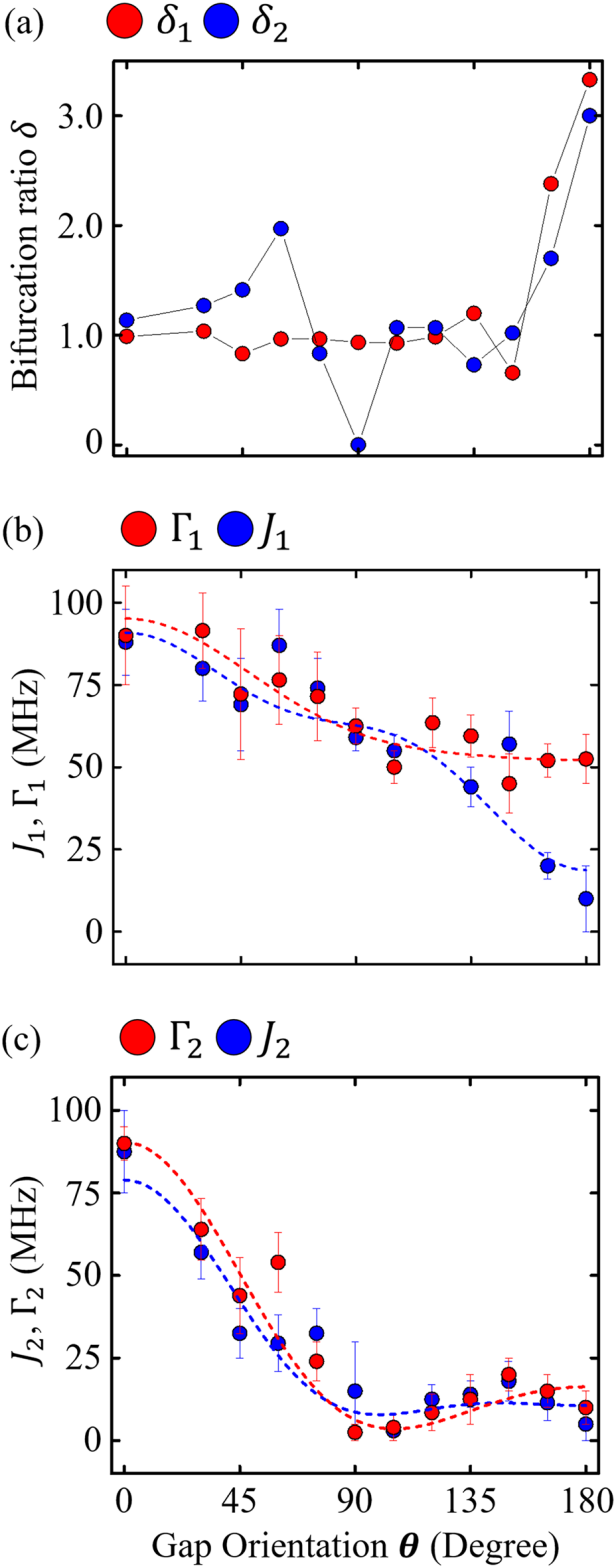
Bifurcation ratios and coupling constants in a dual-photon-magnon hybrid structure based on parameters obtained from measured dispersions and analytical model. (**a**) Experimentally determined bifurcation ratios, ${\delta }_{1}$ (red) and ${\delta }_{2}$ (blue) as functions of the gap angle orientation *θ*. Coupling strengths of (**b**) the lower-branch photon mode and (**c**) the higher-branch photon mode, each interacting with the magnon mode. ${J}_{\text{1,2}}$ and ${\Gamma }_{\text{1,2}}$ indicate coherent and dissipative coupling constants, respectively, with the subscript numbers referring to the two YIG/ISRR hybrids. The scatter points, representing experimental values, include error bars, while the dashed lines indicate theoretical fitting results obtained from a multipolar expansion formula.

A bifurcation ratio, defined as $\delta =\Gamma /J$, serves as a commonly normalized parameter for assessing the dissipation rate in two-level atom systems^26,27,29^, playing a pivotal role in analyzing the dynamics of single photon-magnon coupling^14^. The bifurcation ratios ${\delta }_{1}$ and ${\delta }_{2}$, corresponding to the interaction of each photon mode with the magnon mode, are depicted in Fig. 5a. Despite the linear graph showing similar trends for δ1 and δ2, the photon-photon interaction's impact on these ratios remains subtle with changes in the gap angle orientation $\theta$. However, from $\theta =3\pi /4$ to $\pi$, ${\delta }_{\text{1,2}}$ values show a concurrent rise, signifying an increase in dissipation within the dual-photon-magnon hybrid system, as evidenced by a marked dissipative dispersion, especially when excluding photon-photon interaction considerations.

Figure 5b shows the coupling strengths ${J}_{1}$ and ${\Gamma }_{1}$ between the lower photon hybrid mode and the magnon mode, both of which decrease as $\theta$ increases. Utilizing a fitting equation $f(\theta )=A+Bcos\theta +C{cos}^{2}\theta +D{cos}^{3}\theta$, similar to the photon-photon interaction constant ${g}_{\text{P}}$, we identify that $A$ and $B$ are associated with dipolar excitations, $C$ with quadrupolar, and $D$ with octopolar interactions. The trends, represented by dashed lines for both $J$ and $\Gamma$ in Fig. 5b,c, indicate that the coupling constants exhibit a consistent pattern within the same photon hybrid mode rather than varying according to the type of coupling dispersion. The constants $(A,B,C,D)$ for ${J}_{1}$ and ${\Gamma }_{1}$ are (627, 74.8, -78.8, 286) and (605, 162, 132, 52.5) MHz, respectively, with $A$ being the most significant, highlighting the role of dipolar excitation in influencing the coupling from the lower photon hybrid mode. In contrast, for ${J}_{2}$ and ${\Gamma }_{2}$, the constants transition to (86.3, 102, 362, 240) and (62.8, 218, 471, 152), respectively, showing the dominant effect of quadrupolar excitation, exceeding 20 MHz for both constants. These shifts suggest that the specific photon hybrid mode significantly influences the coupling dispersion in dual-photon-magnon systems, in line with the theoretical expectations.

## Discussion

Our study elucidates the significant influence of photon-photon interactions, particularly within ISRR-ISRR configurations, on the photon-magnon coupling dynamics in dual ISRR/YIG hybrids. This influence is experimentally demonstrated by variations in the relative orientation of the split gaps in these hybrids, leading to the distinctive manifestation of both coherent and dissipative coupling. A comprehensive analytical model substantiates that these unique coupling behaviors stem from the phase differences between the magnon mode and each photon mode, modulated by the relative split-gap orientation. Beyond elucidating the intricate dynamics of photon-magnon coupling, our research paves the way for innovative control mechanisms over these interactions. A prime application of our findings lies in the realm of advanced quantum information processing, where precise manipulation of photon-magnon interactions could play a pivotal role in qubit control and quantum state transfer. Future research directions include exploring innovative photon resonator array configurations and integrating phase shifters to enhance control over these interactions further. Such advancements promise substantial progress in quantum information transfer, leveraging spin degrees of freedom for more efficient and secure optical information processing systems. The potential to extend these insights to more complex systems presents an exciting frontier for future exploration, poised to transform our approach to quantum communication and information processing technologies.

## Methods

### Sample fabrication

The ISRRs were fabricated on a high-frequency laminate substrate known as RF-10, which boasts a relative permittivity of 10. The copper ground plane (25 μm) was etched using photolithography, employing standard printed circuit board techniques. The dielectric and copper layers measure thicknesses of 0.64 mm and 0.035 mm, respectively. The ISRR dimensions are defined as follows: *a* = 3.18 mm, *b* = 2.42 mm, *s* = 0.4 mm, and *g* = 0.4 mm, as depicted in Fig. 1b. Optimization of the ISRRs' dimensions and placements was conducted through numerical simulations with CST Microwave Studio, a commercial electromagnetic full-wave simulator. The microstrip line was designed with a width (*w* = 0.57 mm) to ensure a 50-Ω impedance, determined via microstrip geometry calculations in APPCAD software. The commercial YIG films, deposited on Gadolinium Gallium Garnet (GGG) substrates through Liquid Phase Epitaxy (LPE), share uniform dimensions of 3.7 × 3.7 × 0.025 mm.

### Measurement of S_21_ scattering parameter

The input and output of the microstrip feed line were interfaced with a calibrated vector network analyzer to record the transmittance S_21_ coefficient spectra, as illustrated in Fig. 1a. The S_21_ parameter was assessed in relation to the alternating current (AC) frequency (${f}_{\text{AC}}=\omega /2\pi$) traversing the microstrip line, alongside a DC magnetic field (**H**_*DC*_) sweep applied to the sample. The orientation of the applied DC field, relative to the x-axis (perpendicular to the microstrip line), was adjusted to a critical angle $\phi ={33}^{\circ }$. At this angle, spin wave modes with non-zero wave numbers are absent in the spectra, showcasing exclusively the ferromagnetic resonance (FMR) mode^36^.

### Supplementary Information

Supplementary Information.

## Data Availability

Data supporting this study's findings are available from the corresponding author upon reasonable request.
