# Supplementary material for: Anomalous coherent and dissipative coupling in dual photon-magnon hybrid resonators
Source: Sci Rep. 2024 Jun 12;14:13581. doi: 10.1038/s41598-024-64315-x (PMC11169561; doi:10.1038/s41598-024-64315-x)

**SUPPLEMENTARY MATERIALS**

**Anomalous coherent and dissipative coupling**

**in dual photon-magnon hybrid resonators**

Haechan Jeon, Bojong Kim, Junyoung Kim, Biswanath Bhoi, and Sang-Koog Kim^a)^

*National Creative Research Initiative Center for Spin Dynamics and Spin-Wave Devices,*

*Nanospinics Laboratory, Research Institute of Advanced Materials,*

*Department of Materials Science and Engineering, Seoul National University, Seoul 08826, Republic of Korea*

**S1. Effect on phase difference** $\boldsymbol{\phi}_{\boldsymbol{M}\boldsymbol{1}}$ **and** $\boldsymbol{\phi}_{\boldsymbol{M}\boldsymbol{2}}$ **on multi-photon-magnon coupling**

Previously, as depicted in Fig 2(a), we defined the phase differences between the two-photon hybrid modes and the magnon mode as $\phi_{M1}$ and $\phi_{M2}$, respectively. To verify the impact of each photon hybrid mode on multi-photon-magnon coupling, we examined the changes of magnon damping by varying $\phi_{M1}$ and $\phi_{M2}$ in $\pi/4$ increments from 0 to $\pi$ (Fig. S1). Fig S1(a) presents the result of modifying $\phi_{M2}$ with $\phi_{M1}$ fixed at $\pi/2$. Our analysis revealed that the regions of *θ* forming the dissipative coupling shift according to $\phi_{M2}$. Specifically, when $\phi_{M2}$ is set to zero, anti-damping manifests within a singular area near $\theta=\pi/2$. Conversely, when $\phi_{M2}$ reaches $\pi/2$, anti-damping is observed in two distinct areas at $\theta=\pi/2$ and π. This observation aligns with our previous explanations that $\phi_{M2}$ represents the phase difference between the higher-order resonance mode (over quadrupole mode) arising from the photon-photon interaction and the magnon mode. Therefore, shifts in photon-photon interaction influence the overall dispersion of photon-magnon coupling. In contrast, modifications in $\phi_{M1}$, as shown in Fig 4(b), do not induce notable dispersion changes, although we can observe alterations in the relative strength of damping. This behavior of $\phi_{M1}$ coincides with the physical meaning of the phase difference between the photon base mode and the magnon mode, reflecting its specific influence on the coupling dynamics. This calculation helps us indirectly assess the direct influence of photon-photon interactions on photon-magnon coupling.

**Supplementary Figures**

**FIG. S1.** Effect of the phase difference between each photon hybrid mode and magnon mode. (a) the effect of $\phi_{M2}$ when the$\phi_{M1}$ set $\pi/2$, (b) the effect of $\phi_{M1}$ when the$\phi_{M2}$ set $\pi/2$

**Fig. S1.**


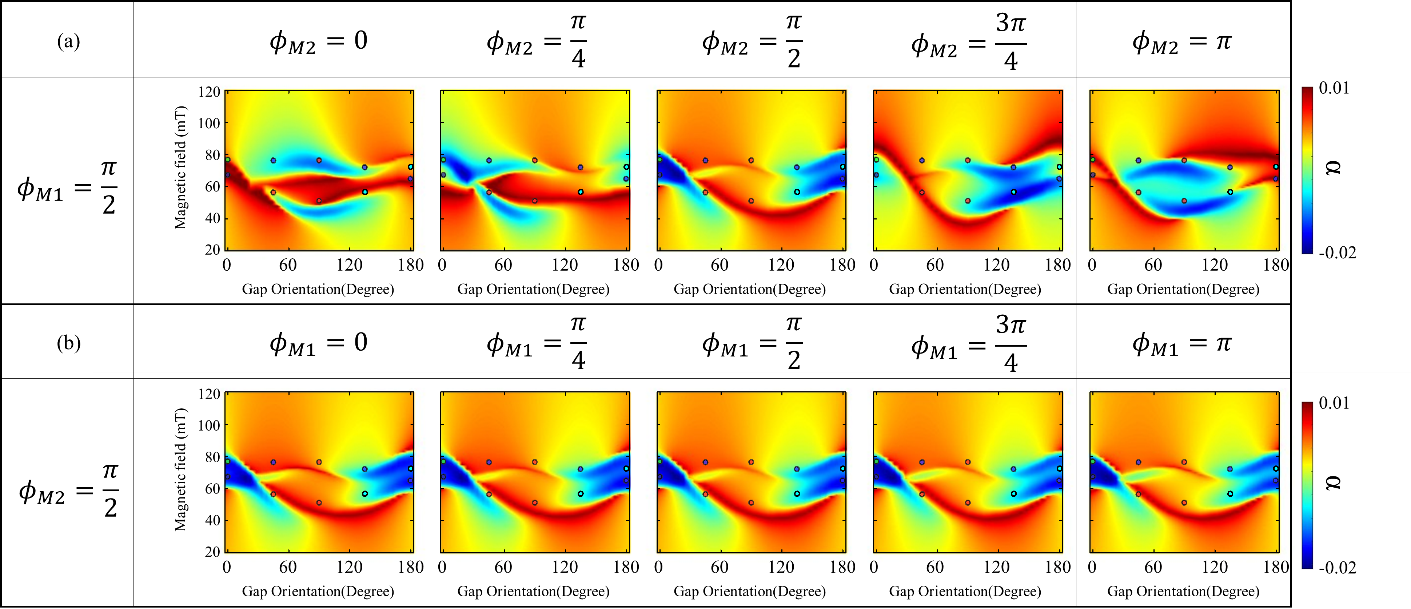

Supplement: Supplementary file 1 — Supplementary Information. [file 41598_2024_64315_MOESM1_ESM.docx]
